# The impact of the human development index on stomach cancer incidence in Brazil

**DOI:** 10.3332/ecancer.2023.1552

**Published:** 2023-05-22

**Authors:** Ana Elisa de Oliveira, Gisele Aparecida Fernandes, Diego Rodrigues Mendonça e Silva, Maria Paula Curado

**Affiliations:** 1 Postgraduate Program in Sciences, AC Camargo Cancer Center, São Paulo, SP 01508-010, Brazil; 2 Pharmacy Course, School of Health Sciences, University of Vale do Itajaí – UNIVALI, Itajaí, SC 88302-901, Brazil; 3 Group of Epidemiology and Statistics on Cancer, AC Camargo Cancer Center, São Paulo, SP 01508-010, Brazil; 4 Hospital Cancer Registry, ACCamargo Cancer Center, São Paulo, SP 01508-010, Brazil; 5 Postgraduate Program in Epidemiology, School of Public Health, University of São Paulo, São Paulo, SP 01246-904, Brazil

**Keywords:** gastric cancer, incidence, epidemiology, socioeconomic conditions, trends

## Abstract

**Background:**

The incidence of stomach cancer (SC) is declining in most countries in the world, potentially associated with increases in the human development index (HDI). This study was conducted to characterise the incidence and trends of SC in the Brazilian population and its correlations with HDI components: longevity, education and income.

**Methods:**

Data on incidence of SC from Population-based cancer registries (PBCR) in Brazil during the period 1988–2017 were extracted from the Instituto Nacional de Câncer. Incidence rates were estimated for each PBCR in the same calendar period. Trends were analysed using the Joinpoint Regression Program, and correlations with HDI components (longevity, education and income) were examined using the Pearson test.

**Results:**

SC incidence rates in Brazil ranged from 22 to 89/100,000 among men and from 8 to 44/100,000 among women. The highest incidence rates for men and women occurred in northern Brazil. The SC incidence is stable in most of the capitals of the northern and northeast parts of the country, with reductions for both sexes in the South, Southeastern and Midwest. There was an inverse correlation of SC incidence rates for women with the components of HDI education (*p* = 0.038) and longevity (*p* = 0.012). For men, the inverse correlation occurred for the longevity HDI (*p* = 0.013).

**Conclusion:**

The improvement of HDIs in Brazil during the study period may have contributed to the stability of SC incidence but was not sufficient to reduce the overall SC incidence in the whole country. To better understand SC incidence in Brazil, efforts should be made towards ensuring that incidence data is recorded by PBCRs promptly.

## Introduction

Stomach cancer (SC) is the fifth most incident cancer and the fourth leading cause of cancer-related death in the world [1]. For men, the incidence of SC occupies the fourth position and the seventh position for women [1–3]. The incidence of SC is declining worldwide [2–5]. In Brazil, the estimated number of SC ranks fourth among men, with 13,360 cases, and sixth among women, with 7,870 cases, representing 5.9% and 3.5%, respectively, of the 10 most common cancers [6]. The incidence of SC among men is highest in northern and northeastern Brazil [6].

The trends of SC incidence and mortality vary among regions of Brazil. Data from three population-based cancer registries (PBCRs) revealed that Belem (Amazon region) had the highest rates and an increasing trend for women, whereas São Paulo (Southeastern) and Fortaleza (Northeastern) showed trends of incidence reduction among men in the period de 1990 a 2012 [7].

SC has multiple risk factors for incidence and mortality, and Helicobacter pylori infection is considered to be the main cause of gastric cancer [3, 8] with a prevalence of 50% in the world population [9] and in Latin America and the Caribbean, before the year 2000, the prevalence was 62.8% [10]. The consumption of salted, smoked and processed foods; excessive alcohol consumption; smoking; gastroesophageal reflux disease; obesity and genetics are also considered risk factors associated with SC [3, 8, 11]. Increased intake of fresh fruits and vegetables and reduced consumption of salty foods, and the availability of refrigeration and good sanitary conditions, can contribute to the reduction of SC incidence [10, 12, 13].

The SC incidence and mortality vary geographically according to the human development index (HDI) [4]. Brazil showed 47.5% HDI growth between 1991 and 2010, when it peaked at 0.727. The country’s HDI has shown improvement since 2000, with an average value of 0.612 [14]. In 2010, the HDI was highest in the southeast of Brazil (0.766), followed by the Midwest (0.756) and South (0.754), whereas HDIs were lowest for the northern and northeastern regions (0.667 and 0.663, respectively) [15] The HDI is a socioeconomic indicator based on three basic dimensions of development: income (wage), education and health (via longevity). SC incidence and mortality are associated with socioeconomic factors such as early access to diagnosis for incidence and treatment for mortality, which improves survival [4].

In Brazil, data on cancer incidence is collected by PBCRs in all regions of the country [16]. This database is crucial for understanding the impact of cancer on the population. This study was conducted to examine the incidence and trends of SC and their correlations with the HDI components: longevity, education and income in Brazil.

## Methods

This is an ecological study, data on the incidence of SC in Brazil during the period 1988–2017 were collected from the PBCR [16]. All cases coded as C16 malignant neoplasm of the stomach were included according to International Classification of Diseases for Oncology, third edition, 2000). The cases were stratified by sex, 5-year age groups from 30 to 34 years to 85 years and over. Capitals that do not have PBCRs were excluded from the analysis (Rio de Janeiro, São Luis, e Macapá); also, PBCRs with a historical series of less than 5 years were excluded from the study (Maceió e Porto Velho). Population data for the period 1988–2012 were obtained from the Unified Health System [17], and from 2012 onwards, Brazilian Institute of Geography and Statistics (IBGE) population projections were used. Age selection from 30 years old was due to the tendency of CS diagnosis.

Crude and age-standardised incidence rates (ASIR) of SC were truncated at the age of 30–85 years. The age-standardised rates incidence was adjusted according to the standard world population by Segi [18].

Trends in the SC incidence rates were analysed using the Joinpoint Regression Program (version 4.7.0.0) [19] and annual average percentage changes (AAPCs) was calculated, when negative AAPCs indicate decreasing trends, positive AAPCs indicate increasing trends, and non-significance indicates stability.

The HDI data were obtained from the United Nations Development Program for the period 2010 [20]. The HDI is a socioeconomic indicator that assesses three basic dimensions of human development in aggregate: income (IDHrd), access to education (IDHed) and health based on longevity (IDHlg), [14, 21]. The IDHrd is the index based on gross national income per capita, IDHed on mean and expected years of schooling, and IDHlg is the index is based on life expectancy at birth, resulting in the average number of years that people would live from birth, considering the same mortality patterns observed in the reference year [15, 21].

To examine the changes in SC incidence, we performed analyses: (1) correlation between the HDI (2010) and age-standardised SC incidence (2) correlation between the HDI (2010) and AAPC. Rates and trends were calculated for a 10-year historical series, from 2006 to 2015, when possible. Considering that the lower the correlation is, the lower the inequality in the rate trend. Both analyses used the Pearson correlation test. The correlation and trend analyses were conducted using STATA 15 [22], with the significance level set to *p* < 0.05. Graphs were generated using Microsoft Excel^®^ (2010), and the *R*^2^ value and line equations were displayed from the linear trend line.

## Results

For all capitals in Brazil, the HDI increased in 2010 in comparison with 2000, with an average increase of 0.11 (range, 0.06 (Porto Alegre) to 0.25 (Boa Vista)).

**Figure 1.**
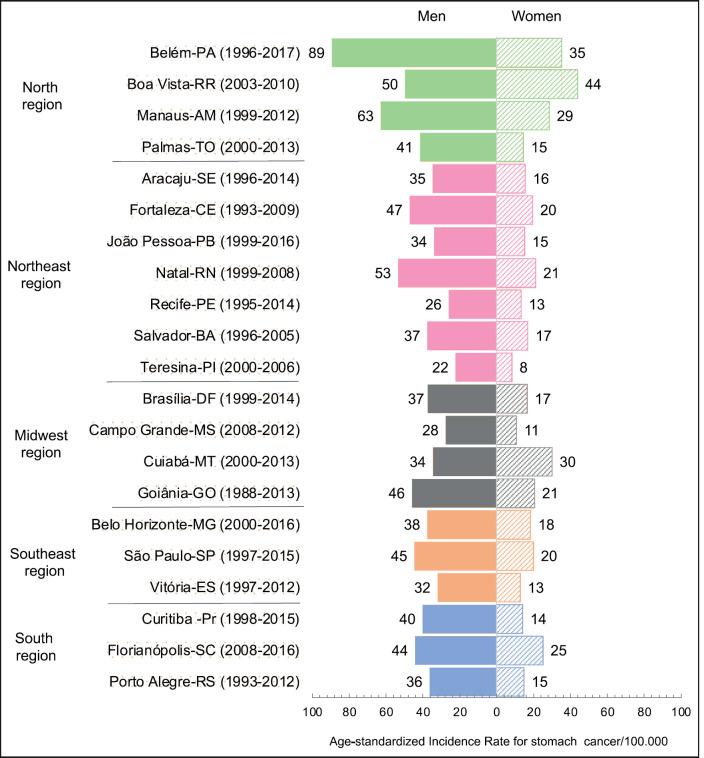
ASIR of SC among men and women in 22 Brazilian capitals (PBCRs).

The age-standardised SC incidence rates in the 22 PBCRS ranged from 22.2 to 89.3/100,000 for men and from 8.4 to 35.3/100,000 for women, being almost three times higher in men. These rates were highest in northern Brazil for Belem-PA, 89.3/100,000 for men, 35.3/100,000 for women (Figure 1, Table 1).

Overall, we identified a trend toward the stability of the SC incidence in Brazil, in men in 14 capitals and 17 capitals among women. A declining incidence trend was observed among men and women in the southern and southeastern regions while the trend was stable for most capitals in the North, Northeast and Midwest for both sexes (Table 1).

The SC incidence rate correlated inversely with the 2010 HDI (*p* = 0.022 for men and *p* = 0.014 for women). While HDI did not correlate with AAPCs for men (*p* = 0.382) and women (*p* = 0.706). However, there was a correlation in the incidence rates between the longevity HDI (IDHlg) for men (*p* = 0.013) and women (*p* = 0.012). The incident rate of SC in women showed an inverse correlation with the HDI of access to education (IDHed) and IDHlg longevity (Table 2).

## Discussion

The incidence of SC in the Brazilian population showed that the highest incidence rates occurred in regions with the lowest HDIs. For men and women, a declining trend was observed in most state capitals in southern and southeastern Brazil, whereas a trend toward stability was observed in the north of the country. However, improvement in the HDI did not correlate with the reduction of the SC trends incidence rate in all capitals of Brazil. It was observed that the lower rates of SC in women correlated with the higher education and longevity. Therefore, in addition to (greater longevity) the educational level may have favored the lower incidence in women. The income did not appear to influence this correlation for women; it may be due to better sanitary conditions due to policies adopted by the government. In 2021, among women aged 15 or over, literacy was 94.5% but 94.1% in men [23].

SC incidence rates were higher among men than women and this profile is already recognised worldwide. The possible explanation includes the fact that men are more exposed to risk factors such as smoking and alcohol beverage consumption, which are associated with SC [24, 25]. The low incidence rates in women may also suggest that estrogen likely protects against SC development [24, 26]. Nevertheless, trends toward stability for both sexes were observed in Brazil, like those observed in some parts of the world [1, 24]. Therefore, improvement in HDI in Brazil did not lead to a decline in incidence rates in the period probably because of the long term exposure to risk factors with no reduced progression of carcinogenesis.

The stability in SC incidence rates observed in Brazil differs from trends of reduction observed in countries with higher HDI such as in the USA and Japan [2, 21, 24, 27]. It also differs from the trend of SC decline observed in Brazil between 2003 and 2007, being −1.7% for men and −1.0% for women annually [24]. The prevalence of *H. pylori* infection is about 57.5% in Latin America and the Caribbean [10], but approximately 71% in Brazil [9, 2]. Despite increases in the HDI a high prevalence of *H. pylori* and lack of information about treatment of this infection in the population may be a factor contributing to the stability of SC incidence rates for most capitals in the North, Northeast, and Midwest for both sexes.

Helicobacter* pylori* infection can contaminate drinking water and vegetables [9, 28]. Countries with high rates of CS (non-cardia) have a high prevalence of *H. pylori* infection, but in developed countries where there has been a reduction in *H. pylori* prevalence, the incidence of CS (non-cardia) has decreased [24]. Thus, control of *H. pylori* infection is an important factor in the control of non-cardia CS in Brazil. However, we could not stratify the data according to SC (cardia and non-cardia) due to a lack of this classification in the PBCRs database.

Sanitation data in Brazilian states in the year 2022 by the Instituto Brazil show that the best basic sanitation is in the municipalities of the South and Southeast and the worst in the North region, some in the Northeast and Rio de Janeiro [29]. These results may explain the findings of this study, such as the downward trend in the incidence of gastric cancer in the Southeast, South and Midwest regions and the high rates in the capitals of the North region

A limitation of this study is related to the fact that the Brazilian PBCRs have no incidence data for the same calendar period. However, this study allows us to describe SC incidence in Brazil and the results suggest the need for close surveillance in the incidence of SC in all Brazilian capitals. In addition, it showed the limitations in comparing data for all capitals due to lack of time series in the registries for the same period. Furthermore, we could not stratify the data according to SC (cardia and non-cardia) due to a lack of this classification at the PBCRs data base.

The improvement of HDIs in Brazil during the study period may have contributed to the stability of the SC incidence but was not sufficientl to reduce the overall SC incidence in the whole country. The longevity and education components of HDI show inverse correlation with SC in women. To better understand SC incidence in Brazil, effort should be made towards ensuring that incidence data is recorded by the PBCRs in a timely manner.

## Conclusion

The incidence of SC showed disparities in rates, probably due to the variation between regional HDIs. Although there was an increase in the HDI in Brazil it did not impact on the reduction of incidence rates in a global fashion. The longevity component was associated with a decrease in incidence, and the education component had more influence in lowering incidence amongst the women than amongst the men. More studies are needed to explore the HDI components to better understand the influence of education in SC cancer incidence in Brazil.

## Conflicts of interest

The authors declare that there is no conflict of interest that could constitute an impediment to the publication of this article.

## Funding

This research was not funded.

## Figures and Tables

**Table 1.** ASIR and AAPC of SC in Brazilian capitals.

Region	Capitals (PBCR)	Period years	ASIR		AAPC			
			Men	Women	Men		Women	
					AAPC	IC	AAPC	IC
Northern	Belém (PA)	1996–2017	89.3	35.3	−5.3	−5.3–2017	−5.3	−5.3–2017
	Boa Vista (RR)	2003–2014	46.0	16.7	−6.7	−6.7–2014	−6.7	−6.7–2014
	Manaus (AM)	1999–2014	63.3	25.7	0.4	−0.47–2014	8.4	−0.47–2014M
	Rio Branco (AC)	2010–2017	72.6	28.7	−8.7	−8.7–2017C	−8.7	7.9; 3.3
	Palmas (TO)	2000–2017	35.7	14.6	-3.5	−3.5–2017	−3.5	25.5; 13.3
Northeastern	Aracaju (SE)	1996–2016	31.2	14.2	−2.4*	−**4.0;** −**0.7**	−0.7;	2.2; 1.5
	Fortaleza (CE)	1990–2015	43.3	19.8	−1.7*	−**2.3;** −**1.0**	0.2	−0.2; 2015
	João Pessoa (PB)	1999–2016	33.8	15.2	1.6	−0.62–2016	1.5	0.8; 3.8
	Natal (RN)	1999–2008	53.4	21.3	−1.3	−1.3–2008	−1.3	−1.3–2008
	Recife (PE)	1995–2017	26.6	13.3	0.0	−0.03–2017	−0.03	−0.03–2017
	Salvador (BA)	1996–2005	37.5	17.0	−7.0	−7.0–2005	0.4	−0.40–2005
	Teresina (PI)	2000–2006	22.2	8.4	−0.42	−0.42–2006(P	3.1	−0.12–2006(P
Midwest	Brasília (DF)	1999–2017	36.9	17.2	−7.2	−7.2–2017	−7.2	−7.2–2017
	Campo Grande (MS)	2008–2012	27.6	10.9	−0.9	−0.9–2012nd	−0.9	−0.9–2012nd
	Cuiabá (MT)	2000–2016	48.8	24.5	−4.0*	−**6.7;** −**1.2**	−1.3	−0.3; 2016
	Goiânia (GO)	1988–2013	45.8	20.6	−1.4*	−**2.5;** −**0.2**	−2.1*	−**3.5; 0.8**
Southeastern	Belo Horizonte (MG)	2000–2017	40.3	18.5	−2.7*	−**3.7;** −**1.7**	−1.9*	−**3.1;** −**0.7**
	São Paulo (SP)	1997–2015	44.5	20.0	−6.4*	−**7.3;** −**5.5**	−5.1*	−**6.0;** −**4.3**
	Vitória (ES)	1997–2012	31.9	12.8	0.4	−0.48–2012	0.1	−0.18–2012
Southern	Curitiba (PR)	1998–2017	39.8	13.8	−2.7*	−**3.8;** −**1.7**	−4.5*	−**5.5;** −**3.5**
	Florianópolis (SC)	2008–2016	44.1	25.2	−5.2	−5.2–2016	−5.2	−5.2–2016
	Porto Alegre (RS)	1993–2017	33.7	14.2	−3.3*	−**4.2;** −**2.3**	−2.2*	−**3.5;** −**0.9**

IC – Confidence interval 95%

* Statistically significant difference

PBCR – Population-Based Cancer Registries

**Table 2.** Correlations ASIR and AAPC of SC versus HDI-2010.

	ASIR						AAPC					
	Men			Women			Men			Women		
	*R* ^2^	*r*	Value-*p*	*R* ^2^	*r*	Value-*p*	*R* ^2^	*r*	Value-*p*	*R* ^2^	*r*	Value-*p*
**HDI**	**0.305**	**−0.305-**	0.022*	**0.353**	−0.353*	0.014*	0.051	−0.051*	0.382	0.009	−0.009*	0.706
**HDI_rd_**	0.179	−0.179*	0.091	0.193	−0.193*	0.077	0.004	−0.004*	0.806	0.001	0.025	0.923
**HDI_ed_**	0.203	−0.203*	0.069	**0.256**	−0.256*	0.038*	0.078	−0.078*	0.277	0.045	−0.045*	0.413
**HDI_lg_**	**0.345**	−0.345*	0.013*	**0.354**	−0.354*	0.012*	0.035	−0.035*	0.471	0.000	−0.000*	0.960

* Statistically significant difference

Legend: HDI income (HDI_rd_), HDI education (HDI_ed_), HDI longevity (HDI_lg_)
